# Report of the Eastern Lunatic Asylum in the City of Williamsburg, Virginia

**Published:** 1853-05

**Authors:** 


Art. X.—*Report of the Eastern Lunatic Asylum in the city of Williamsburg, Virginia.* 1852-3.

We are indebted to the intelligent Superintendant and Physician, Dr. John M. Galt, for a copy of the report on the condition and prospects of the Eastern Lunatic Asylum of Virginia. The number of patients in the Asylum since the 30th of September, 1851, has been 247. Among these, there have been 16 deaths—12 males and 4 females. At present, the number of patients is 211. The deaths have occurred chiefly among the old and incurable cases, and mostly from diarrhea, which, according to Millingen, "is one of the most frequent and fatal complications of insanity."

"Of the discharges seventeen, ten females and seven males, were under the head of recoveries. Two of the males were very much improved, and indeed returned to their accustomed avocat.ons in life soon after their discharge. In nearly all asylums for the insane, there is such an arrangement as to patients, that there must be left within their confines a mass largely predominating in number, constituting as it were the *debris* of successive years, and which are almost wholly incurable. The number of recoveries then depends pretty much upon the number of receptions during a given time. Thus the distinguished physician of a very large and very celebrated English institution stated in one of his reports, that at the beginning of the official year, the 1st of October, there were 975 patients in the asylum, and that of this large number there were not probably forty curable cases. Whilst the able physician of the Ohio state asylum says in his last report, that at the commencement of the year there were three hundred and one patients in that institution, and that the prospect of recovery in two hundred of them was "very remote." Then again, the character of the receptions materially varies the ultimate result—the comparative number of recent and curable, and of old and incurable cases. Moreover, in some institutions, only a certain class of patients are received. Thus, in two of the London hospitals, all persons were rejected 'who had been insane more than a year, those affected by paralysis and epilepsy, and the aged and feeble.'"

